# Overcoming technological barriers in microfluidics: Leakage testing

**DOI:** 10.3389/fbioe.2022.958582

**Published:** 2022-09-07

**Authors:** Vania Silverio, Suvajyoti Guha, Armelle Keiser, Rucha Natu, Darwin R. Reyes, Henne van Heeren, Nicolas Verplanck, Luke H. Herbertson

**Affiliations:** ^1^ Instituto de Engenharia de Sistemas e Computadores para os Microsistemas e as Nanotecnologias, INESC MN, Lisboa, Portugal; ^2^ Department of Physics, Instituto Superior Tecnico, Universidade de Lisboa, Lisboa, Portugal; ^3^ Office of Science and Engineering Laboratories, Center for Devices and Radiological Health, U.S. Food and Drug Administration, Silver Spring, MD, United States; ^4^ Microfluidic Systems and Bioengineering Lab, Univ. Grenoble Alpes, Technologies for Healthcare and Biology Division, CEA/LETI, Grenoble, France; ^5^ National Institute of Standards and Technology, Gaithersburg, MD, United States; ^6^ EnablingMNT/The Microfluidics Association, Dordrecht, Netherlands

**Keywords:** microfluidics, biomedical devices, leakage testing, standards, pressure decay, mechanical integrity, liquid/gas leak

## Abstract

The miniaturization of laboratory procedures for Lab-on-Chip (LoC) devices and translation to various platforms such as single cell analysis or Organ-on-Chip (OoC) systems are revolutionizing the life sciences and biomedical fields. As a result, microfluidics is becoming a viable technology for improving the quality and sensitivity of critical processes. Yet, standard test methods have not yet been established to validate basic manufacturing steps, performance, and safety of microfluidic devices. The successful development and widespread use of microfluidic technologies are greatly dependent on the community’s success in establishing widely supported test protocols. A key area that requires consensus guidelines is leakage testing. There are unique challenges in preventing and detecting leaks in microfluidic systems because of their small dimensions, high surface-area to volume ratios, low flow rates, limited volumes, and relatively high-pressure differentials over short distances. Also, microfluidic devices often employ heterogenous components, including unique connectors and fluid-contacting materials, which potentially make them more susceptible to mechanical integrity failures. The differences between microfluidic systems and traditional macroscale technologies can exacerbate the impact of a leak on the performance and safety on the microscale. To support the microfluidics community efforts in product development and commercialization, it is critical to identify common aspects of leakage in microfluidic devices and standardize the corresponding safety and performance metrics. There is a need for quantitative metrics to provide quality assurance during or after the manufacturing process. It is also necessary to implement application-specific test methods to effectively characterize leakage in microfluidic systems. In this review, different methods for assessing microfluidics leaks, the benefits of using different test media and materials, and the utility of leakage testing throughout the product life cycle are discussed. Current leakage testing protocols and standard test methods that can be leveraged for characterizing leaks in microfluidic devices and potential classification strategies are also discussed. We hope that this review article will stimulate more discussions around the development of gas and liquid leakage test standards in academia and industry to facilitate device commercialization in the emerging field of microfluidics.

## 1 Introduction

While promising microfluidic technologies are emerging in the biomedical field, standard test methods have not yet been established to validate basic manufacturing steps, performance, and safety of microfluidic devices. The successful development and widespread use of microfluidics are dependent on concurrent standardization efforts in the community. One common issue with microfluidic systems is leakage. Unintended leaking of a microfluidic device can prevent it from functioning properly and create safety concerns. Since there are no commonly accepted means for evaluating leakage in microfluidic device, it is important to establish consensus test plans for characterizing leaks in this type of environment (Reyes et al., 2020). Thus, the objectives of this review paper are to: (1) review existing standards and test methods that are currently used for leak testing and detection in other fields; (2) identify community-wide issues that may occur when microfluidic technologies leak; and (3) encourage the microfluidics community to create new guidelines for reliably assessing leaks.

Leaks can be difficult to detect in a microfluidic environment because of the small fluid volumes involved and because they can occur at the microfluidic component interface with interconnectors, within the microchannel, across the material, or at the upstream or downstream components of the device. Microfluidic devices can be multiplex systems comprised of different materials and components bridged together by connectors, which are further prone to leaks. In microfluidic systems, even a small leak may be catastrophic due to the small total volume of the system, and device performance, like measurement accuracy or output, can be greatly impacted. Therefore, any leakage testing protocol for microfluidic systems should be highly sensitive to detect minute changes in flow.

**FIGURE 1:**
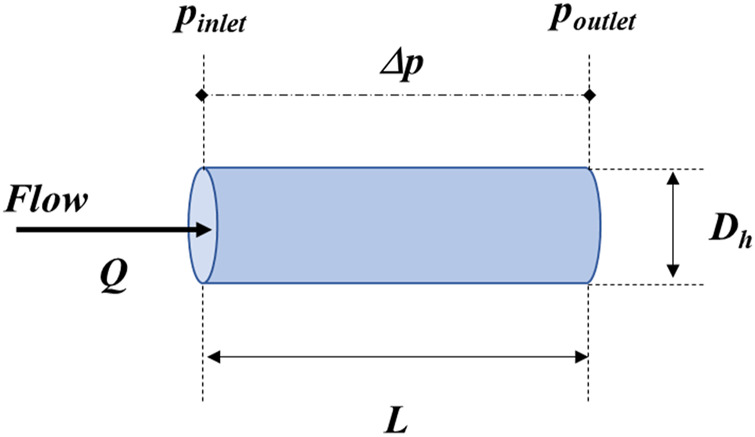
Representation of the geometric and flow parameters of the microchannel corresponding to Eq. 1.

As seen from the Hagen-Poiseuille equation below (Eq. 1) for laminar flow of an incompressible Newtonian fluid in a cylindrical channel with invariant cross-sections (Figure 1):

$$\Delta p=\frac{32\mu LQ}{\pi D_{h}^{4}}$$ (1)

where, ∆p is the pressure drop, μ is the fluid dynamic viscosity, L is the channel length, Q is the volumetric flow rate, and D_h_ is the hydraulic diameter of the channel. Changes in pressure are inversely proportional to the fourth power of the diameter. Therefore, when driving flow through smaller channels higher pressures will be generated, thus increasing the likelihood of leakage. Higher pressure is required to drive flow through smaller channels, which increases the likelihood of leaking. Additionally, leakage measurements and detection modes need to be made and controlled by sensors and instruments capable of operating in the limited space on the microscale level.

It is important to understand how a microfluidic system leak occurs regardless of whether the application resides in the chemical or biomedical field. The focus of this review is on biomedical applications, for which leaking is a potentially catastrophic problem that may compromise the safety and effectiveness of a medical device. For instance, leakage of a diagnostic medical device may result in the loss of blood analytes and the need to repeat a test, which is likely to interfere with clinicians’ ability to make timely healthcare decisions and effectively treat patients. In a therapeutic device, unexpected leakage from a patient-contacting device into the interstitial tissue may pose biocompatibility, leaching, and toxicological safety risks. In a combination product such as a drug delivery system, a leak can prevent the intended drug dose from being delivered to a patient. In situations like these, it is necessary to assess the extent of the safety risk from the leak, identify the location of the leak, quantify the leakage rate using a sensitive detection technique, and subsequently take corrective and preventative actions to resolve the failure mode. Conversely, some amount of leakage may be tolerable in certain microfluidic systems depending on the application. For example, when a liquid must reach equilibrium with a sensor surface to enable a measurement, leaking may not necessarily impact the reading or device performance if enough sample is provided to cover the surface of the sensor. If the leak does not pose a safety threat to the user or environment, it may be sufficient to simply include a leakage specification based on safety and health regulations for maximal exposure (Health and Executive, 2013), clinical assessment, or “Instructions for Use” detailing the implications of an observed leak.

A survey by The Microfluidics Association (The Microfluidics Association, 2021) indicates that many device developers have been using their own in-house protocols for leakage testing and detection for decades, mainly because there are currently no standard protocols to meet this need on the micro-scale. Yet, the need for harmonization of such efforts is of critical importance now that the number of microfluidic-based medical device submissions to regulatory agencies like the U.S. Food and Drug Administration (FDA) is steadily increasing (Reyes et al., 2020). Previously, a survey of more than 100 organizations in the microfluidics space found that of the many adverse events seen by device developers during operation, “leakage” was identified as one of the most common failure modes (The Microfluidics Association, 2015). And the community recognizes that standard test methods for evaluating leakage in microfluidics are imperative throughout the total product life cycle, from prototyping to commercialization.

Consensus-based leakage testing protocols or standards can provide clarity about the mechanical integrity of a system during device development and for quality control assessment. During the development phase, leakage tests can help to identify early design vulnerabilities that may need improvement and to ensure that design and fabrication procedures follow environmental and safety regulations. These iterations can lead to a final version of the product that does not leak during handling and operation. In later production phases, leakage testing continues to be beneficial to ensure that the end-product will perform as designed and all units are safe for their intended use. If leaks are detected in the end-product, corrective actions can be taken to improve the production process and enable the device to meet specifications.

Although a few standards already exist to assess the propensity for larger-scale devices to leak (Table 1), these are not necessarily applicable for microfluidic devices and no consensus test methods have yet been established to evaluate the leakage of microfluidic medical devices. To determine the extent to which existing standards and test methods can be leveraged or modified for microfluidic systems, we have assessed the transferability of those tests to microfluidic regimes.

**TABLE 1:** Examples of existing standards from ASTM International and International Organization for Standardization (ISO) that contain aspects of leakage testing.

Standard number	Title of the standard
ASTM F2391-05:2016	Standard test methods for measuring package and seal integrity using helium as the tracer gas. https://www.astm.org/f2391-05r16.html
ASTM E432-91:2017	Standard guide for selection of a leak testing method. https://www.astm.org/e0432-91r17e01.html
ASTM F2338-09:2020	Standard test method for nondestructive detection of leaks in packages by vacuum decay method. https://www.astm.org/f2338-09r20.html
ISO 10648-2:1994	Containment enclosures—Part 2: Classification according to leak tightness and associated checking methods. https://www.iso.org/standard/20483.html
ISO 27895:2009	Vacuum technology—Valves—Leak test. Reviewed and confirmed in 2020. https://www.iso.org/standard/44418.html
ISO 13056:2011	Plastics piping systems—Pressure systems for hot and cold water—Test method for leak tightness under vacuum. Reviewed and confirmed in 2017. https://www.iso.org/standard/52473.html
ISO 13503-6:2014	Petroleum and natural gas industries - Completion fluids and materials—Part 6: Procedure for measuring leakoff of completion fluids under dynamic conditions. Reviewed and confirmed in 2019. https://www.iso.org/standard/56107.html
ISO 3503:2015	Plastics piping systems—Mechanical joints between fittings and pressure pipes—Test method for leak tightness under internal pressure of assemblies subjected to bending. Reviewed and confirmed in 2020. https://www.iso.org/standard/61260.html
ISO 1135-4:2015	Transfusion equipment for medical use—Part 4: Transfusion sets for single use, gravity feed. https://www.iso.org/standard/63425.html
ISO 18081:2016	Non-destructive testing—Acoustic emission testing (AT)—Leak detection by means of acoustic emission. https://www.iso.org/standard/61326.html
ISO 7199:2016	Cardiovascular implants and artificial organs—Blood gas exchangers (oxygenators). https://www.iso.org/standard/67607.html
ISO 20485:2017	Non-destructive testing—Leak testing—Tracer gas method. https://www.iso.org/standard/68190.html
ISO 20486:2017	Non-destructive testing—Leak testing—Calibration of reference leaks for gases. https://www.iso.org/standard/68191.html
ISO/AWI 8639, 2017	Glass-reinforced thermosetting plastics (GRP) pipes and fittings—Test methods for leak tightness and proof of structural design of flexible joints. https://www.iso.org/standard/84121.html
ISO 13259:2020	Thermoplastics piping systems for underground non-pressure applications—Test method for leak tightness of elastomeric sealing ring type joints. https://www.iso.org/standard/80706.html

## 2 Types of leakage testing

### 2.1 Leakage test media

#### 2.1.1 Gas-based testing

Traditionally, macroscale gas-based leakage detection methods employ different external and internal sensing techniques than liquid-based testing. External techniques include visual methods such as observing unwanted air bubble formation, system pressure decay, noise, or vibrations (Sagi, 2001; Adegboye et al., 2019; Zhang et al., 2019), whereas internal sensing methods include monitoring pressure, flow rate, temperature, and density within the microchannel to quantitatively characterize the unintended release of product from the microfluidic system (Vtech, 2006; Real Zero, 2009). Monitoring parameters such as mass-volume balance, negative pressure waves, and pressure point analyses often require measurements at multiple locations along the flow path. Leak rates can be detected with an uncertainty of less than 10% using these gas-based techniques (Zaman et al., 2019). This 10% uncertainty is based on factors such as detection method, measurement resolution and accuracy, channel dimensions, position along the channel, and properties of the media used. Gas-based testing for leakage is described in detail in DIN EN 1779:1999 (German Institute for Standardization, 1999). The standard specifies factors such as time dependence, flow conditions (e.g., viscous laminar or molecular flow), pressure, temperature, nature of gas, and cleanliness of the test unit. DIN EN 1779:1999 also describes the influence of these factors on leakage and criteria for selecting the appropriate leakage testing method. In general, testing with gases is non-destructive and suitable for high volume production (Sagi, 2001). Testing with gases involves first connecting the device under investigation to a pressure source and a sensitive manometer. All downstream ports are closed, and the system is pressurized to a worst-case condition or 1.5 to 2 times the maximum expected pressure. Then, the pressure source is disconnected, and pressure decay is monitored as a surrogate for device leakage.

Manufacturers sometimes develop customized leakage testing protocols to accommodate the features and requirements of their specific microfluidic systems. For example, one test facility provides a two-step process using compressed air, nitrogen, or vacuum to detect leaks and blockages in microfluidic channels (Cincinnati Test Systems, 2020). This method utilizes pressure decay for leakage detection, though the precise location of any leak is not determined. Elsewhere, an air leakage detection system detects leak rates in low flow devices with a resolution as small as 7 × 10^−5^ Pa L/s (< 1 μ m defect size; Vacuum, 2018). The detection method employs flow, pressure, and temperature measurements to provide an output that is directly proportional to the leak rate and defect size. This detection system can be used in micro-electromechanical systems (MEMS) devices and biomedical applications for traceable flow rates as low as 1 μ L/min. Antelius, et al. (2011) used another vacuum system to detect leaks as small as 1.4 × 10^−8^ Pa L/s (hole diameter 24 μ m, noise level of the detector 1 × 10^−8^ Pa L/s) in a MEMS-based hermetic liquid integration device fabricated on a silicon wafer. Depending on the detection method used, finding the exact location of a leak may be difficult. For example, soapy water can be used to help locate the compromised area.

For a cyclic olefin copolymer (COC) nebulizer microfluidic chip design, Sen and DarabiKnapp. (2009) connected the capillaries to a syringe pump to push air through the system at a moderate pressure and observe for bubbles. For this type of test, it is critical to use an appropriate applied pressure, and the integrity of the design is heavily dependent on the interconnections to the chip. Liu and Li (2014) reported a procedure used for coarse and fine leak testing of an internal electromagnetic valve in a polydimethylsiloxane (PDMS)-based microfluidic device. The coarse testing was performed by flowing nitrogen through the closed microfluidic device submerged in a water bath and detecting bubble formation. The gas pressure was increased from 0 kPa to 280 kPa - a value that was 1.4 times higher than the 200 kPa operating pressure of the channel. Using a flow rate sensor, the system was able to detect a leak rate of 3.1 × 10^−7^ kg/s (0.026 sccm or standard cubic centimeters per minute) at a pressure of 200 kPa through the microvalve. For an implantable microfluidic intraocular pressure sensor sealed with parylene-C proposed by Araci et al. (2014), gas leakage testing through the sensor walls was conducted over 3 days to characterize changes in leakage over an extended period. From these examples it is evident that gas-based leakage testing can be effectively used to characterize the mechanical integrity of different types of microfluidic devices and components. Yet, it is not clear what the allowable leakage rate requirements should be for these devices, and the impact of the microchannel material on leakage is not well understood. In all cases, the pressure range of the testing and the maximum test pressure should be justified based on specific application requirements.

When using gas-based leakage methods, the correlation between the gas leakage rate and that of the actual intended medium must be known to accurately interpret the leakage test results. In general, an air-based leak test produces 51 times the amount of leaked medium compared to water, assuming that the difference is governed only by viscosity (Amesz, 1966). However, this correlation has not been validated for small leaks expected in microfluidic devices. Although gas permeation can be monitored in PDMS membranes (Yang and Kao, 2010; Limitless Shielding, 2020), gas-based leakage testing may not be suitable when permeable materials of construction like PDMS are used, or in cases where the leakage is influenced by the interactions between the device materials and the flowing medium. For instance, it may be necessary to test with the actual intended medium to assess delamination caused by the interactions between organic solvents and device interfaces. An additional consideration is the sensitivity of the detection method. Since the internal volume of the test setup contributes to the sensitivity of the manometer, the test setup should be optimized by using a small internal volume, with short tubing segments of small cross-sectional area and low-volume connectors. To ensure that expansion and compression of device components (e.g., compliant tubing) do not adversely impact the test results, stainless steel tubes should be used for gas-based leakage testing when possible. For ease of use and to prevent damage to the device, leakage tests should be done with dry air or nitrogen. Also, gas-based techniques can be adapted for determining the bonding strength of microfluidic device connections (Xu et al., 2012; Yin and Zou, 2018).

#### 2.1.2 Liquid-based testing

While much work has been done at the macroscale using gas-based leakage detection methods to define leak testing specifications (Table 1), liquid-based leakage testing is less common for quality control assessment due to the contaminative and destructive nature of these tests. In general, similar to gas-based detection, small liquid leaks can be quantified through mechanical integrity tests such as pressure decay, and global flow measurements can be made to characterize larger leaks. These methods typically rely on optical visualization of the leak itself or measurements of unintended pressure changes. Pressure measurement techniques also often involve wetting the fluid-contacting surfaces, filling the device to be tested with the test fluid and displacing all air out of the system, connecting the system to a pressure source and a manometer, using valves or clamps to close the system, pressurizing the system to a specified pressure (i.e., 1.5 to 2 times the maximum operational or labeled pressure), and then disconnecting the pressure source and monitoring for any pressure decay.

Visualization techniques are the simplest, least burdensome, and most common methods for determining fluid leaks of devices routinely used at home or in situations where qualitative estimates are acceptable. However, in an R&D or a manufacturing setting, visualization methods often involve filling the device under investigation with the test fluid to simulate worst-case operating conditions, in terms of pressure, flow rate, and temperature, for the intended duration of use of the device. The test duration may last several minutes to hours or even days depending on the measurable leakage rate and the intended duration of use. The system is monitored at regular intervals or over the entirety of the test, and the device fails the leakage test if liquid is observed outside the flow path. More rigorous observations and inspections can be made in microfluidics using a camera and microscopy over a range of wavelengths because of the planar attributes and optical transparency commonly associated with these devices. The overall uncertainty of the measurement is highly dependent on the resolution of the detection apparatus, as well as factors such as liquid evaporation and test duration (Schroder, 2001; Kauffman et al., 2010; Ogheard et al., 2020; Batista et al., 2021). Color dyes (Shiroma et al., 2016) or fluorescent tracers (Chueh et al., 2007; Trietsch et al., 2017; Gonzalez-Gallardo et al., 2021) are often added to the test fluid to easily monitor the flow pathway and, in turn, any leaks that may occur.

**FIGURE 2:**
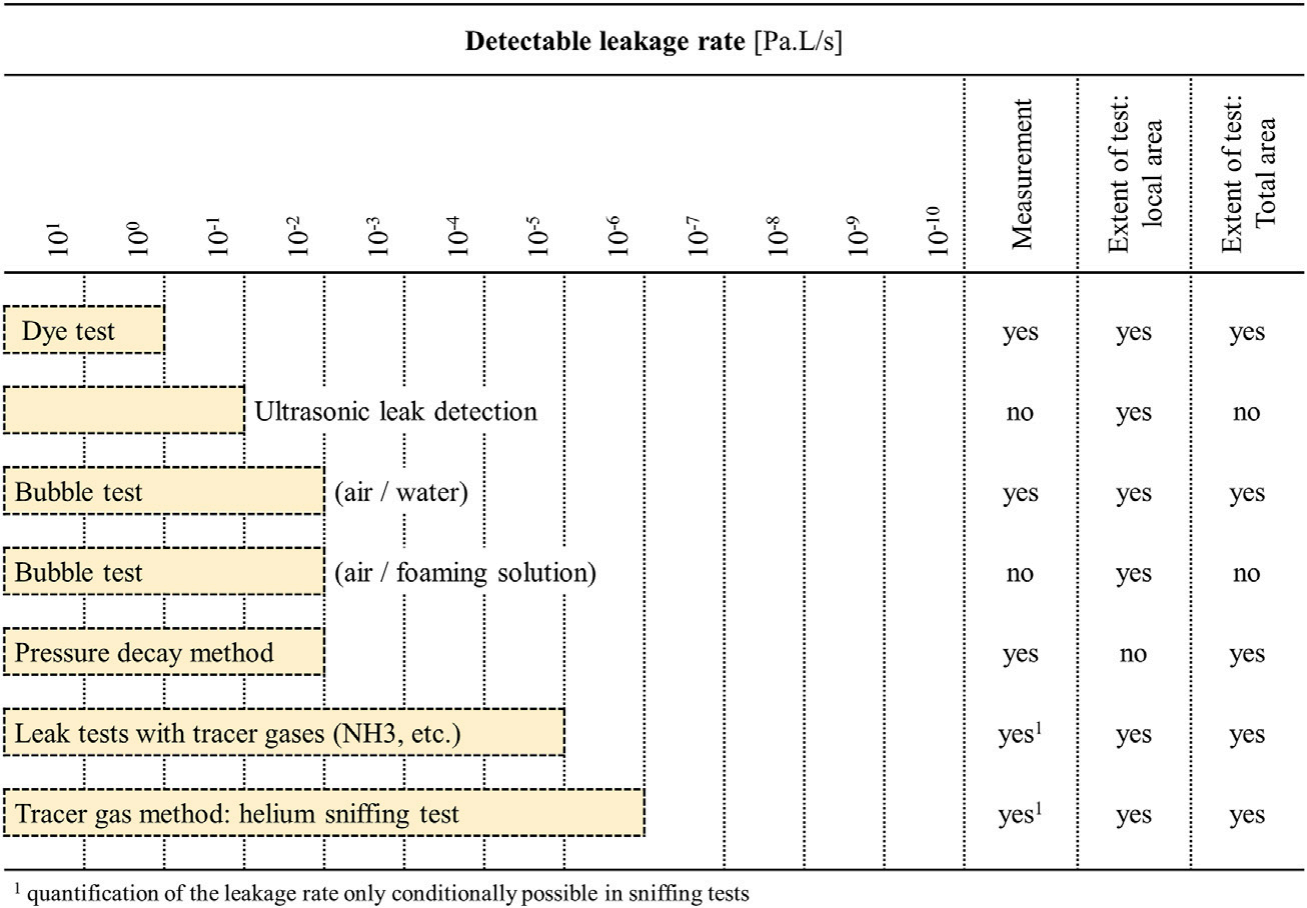
Types and detection limits of various leakage detection methods, in terms of changes in pressure over time. Adapted from Schroder (2001).

Figure 2 shows common liquid- and gas-based leakage test methods arranged by increasing test sensitivity (Schroder, 2001). Dye based liquids are often the easiest to implement but also the least sensitive. Wettability of the liquid-surface combination, the presence of sharp corners that can promote bubble formation, a long channel length resulting in increased pressure drop, capillary effects, and surface tension of the liquid can all contribute towards making liquid testing ineffective or largely dependent on the channel design (Oosterbroek et al., 2005; Vowell, 2009; Wang et al., 2010).

Pressure decay testing to identify leaks typically requires placing pressure sensors at the inlet and/or outlet of the microchannel (Casanova-Moreno et al., 2017). A reproducible method to characterize microfluidic channel deformations and liquid leaks can be achieved by positioning a sealed capillary tube with a calibrated scale at the channel outlet (Shen et al., 2020). The resolution of the measurement can be improved by simply decreasing the capillary diameter, further resolving the scale, or by complementing the visualization technique with optical detection of the fluid meniscus. In a well-defined system, the pressure results can be compared with a theoretical analysis of the flow. High occurrences of blockages or leaks may be indicative of physical defects in a microfluidic system. Hu, et al. (2014) modeled the microfluidic flow paths and flow control, consisting of pumps and pressure sensors, as a logic circuit composed of Boolean gates. The true pressure sensor measurements from the microfluidic system could then be compared to expected outputs from the idealized logic circuit model, which supports the identification of types and locations of any physical defects.

While liquid-based testing can be a simple and effective way to assess microfluidic device leakage, there are some inherent disadvantages associated with this type of testing. For instance, liquid-based pressure measurements can be labor intensive. If the device is intended to be a single-use assay or consumable, liquid-based testing is often destructive such that the device cannot be used again after the leakage test has been performed. While pressure decay liquid-based leakage testing is usually more sensitive than visual inspection for leakage, pressure measurements assess the system in its entirety. Therefore, it may be difficult to determine the location of any leaks that occur while using techniques based on pressure measurements, even if the extent of the leakage can be more readily quantified. In contrast to pressure measurements, visualization-based techniques tend to be more qualitative than quantitative, and visualization-based techniques can only be used with nontoxic or nonhazardous liquids or surrogate liquids. Also, only substantial leaks can be detected visually, as the smallest observable volume is a droplet of a few microliters. Using a dye or colored liquid and wiping the outer surface with a dry cloth before testing can increase the sensitivity of the visualization test. However, these procedural steps may potentially compromise the functionality of the device. Despite these limitations, visualization-based techniques are often used to identify design flaws based on the location where leaks occur.

### 2.2 Materials of construction

In addition to understanding the flow restraints of microfluidic devices, it is important to recognize that a wide variety of materials are used in the microfluidic industry. Commonly used materials in the construction of microfluidic components and devices are presented in Table 2. The material and surface properties are expected to affect how a device leaks (e.g., bonding quality, surface tension, absorption), but there is a lack of fundamental studies in this area. Information about common materials and their compatibility for microfluidic applications can be found in Table 3 and is further reviewed in Roy et al. (2016). Biomedical microfluidic systems often come into short- or long-term contact with different fluids (e.g., water, buffers, organic solvents, blood) and surfaces (e.g., skin, syringes) during sample loading, user handling, and device operation. The microfluidic materials used will impact the location and mechanism by which a leak may occur. Elastomers are flexible materials with low creep resistance. Thermoplastic elastomers can melt, whereas thermosetting elastomers do not melt. Examples of elastomers used in microfluidics are synthetic rubber, which includes EPDM (ethylene propylene diene monomer) rubber, FFKM (perfluorinated elastomers), and silicones, of which PDMS is widely used. Although PDMS is a very popular material in academic research settings due to its ease for prototyping and fabricating low quantities of units during the research and development phases of the product life cycle, scalability challenges and cost prevent PDMS from being a preferred industry choice for mass production of microfluidic devices (Mukhopadhyay, 2007). There is a stronger preference by industry to use materials like COC, COP (cyclic olefin polymers), glass, and PMMA (polymethylmethacrylate) to create highly resolved, reproducible microfluidic structures. Glass is often used for some of the most demanding applications, such as reusable or long-term devices or those operating at high pressures and temperatures. While COC and COP are mainly used for low-cost disposables in point-of-care (POC) applications, POC devices can still be composed of glass due to its relatively high accuracy, short “time to result”, and its ability to be integrated with electronic components (Salvo and Hernandez-Silveira, 2016). Other materials such as polycarbonate (PC) and polystyrene (PS) are popular choices for cell culturing and OoC applications. Regardless of the primary material used, no single material is suitable for all subcomponents within the most complex microfluidic devices (Silverio and Cardoso, 2021). It is not uncommon for several different materials to be used in a microfluidic system, and more interconnections and material interfaces increase the risk of leakage. The bonding method used, material degradation, and deformation can also cause a device to leak. In addition to the common microfluidic materials mentioned above, niche areas exist in microfluidics which employ other materials. For instance, the popularity of 3D printed microfluidic systems is growing, but technical constraints and high costs currently limit the widespread use of 3D printing for microfluidic applications. Additionally, high pressure microfluidic applications may require stronger components comprised of stainless-steel or stainless-steel variants.

**TABLE 2:** Commonly used materials in microfluidics [Adapted from (The Microfluidics Association, 2015)].

Components	Materials
Chips, substrates	COC/COP, PMMA, PC (polycarbonate), PS, silicon, glass
Tubing	PEEK (polyether ether ketone), FEP (fluorinated ethylene propylene), silicone, PTFE (polytetrafluoroethylene)
Connectors	PEEK, PTFE, FFKM
Reservoirs, pouches, blisters	PEEK, COC and PP (polypropylene)
O-rings, gaskets	Rubber
Electrodes	Metals (e.g., platinum, gold)
Sensors	Metals, oxides (e.g., silicon oxide), nitrides (e.g., silicon nitride) *in the case of biosensors, often with functionalized surfaces*

**TABLE 3:** Overview of properties and applications of materials commonly used in microfluidics (adapted from Ren et al., 2013).

	Silicon/Glass^a^	Elastomers	Thermoset	Thermoplastics	Hydrogel	Paper
PROPERTY						
Young’s (tensile) modulus (GPa)	130-180/50-90	0.0005	2.0–2.7	1.4–4.1	low	0.0003–0.0025
common technique for microfabrication^b^	photolithography	casting	casting, polymerization	thermomolding	casting, photopolymerization	photolithography, printing
smallest channel dimension	< 100 nm	< 1 mm	< 100 nm	∼100 nm	∼10 mm	∼200 mm
channel profile	limited 3D	3D	arbitrary 3D	3D	3D	2D
multilayer channels	hard	easy	easy	easy	medium	easy
thermostability	very high	medium	high	medium to high	low	medium
resistance to oxidizer	excellent	moderate	good	moderate to good^c^	low	low
solvent compatibility	very high	low	high	medium to high	low	medium
hydrophobicity	hydrophilic	hydrophobic	hydrophobic	hydrophobic	hydrophobic	amphiphilic
surface charge	very stable	not stable	stable	stable	n/a	n/a
permeability to oxygen (Barrer^d^)	< 0.01	∼500	0.03–1	0.05–5	> 1	> 1
optical transparency	no/high	high	high	medium to high	low to medium	low
APPLICATIONS						
CE	excellent	moderate	good	good	n/a	n/a
electrochemical detection	good	limited	moderate	moderate	no	moderate
Organic synthesis	excellent	poor	good	moderate to good	n/a	n/a
droplet formation^e^	excellent	moderate	good	good	n/a	n/a
PCR	excellent	good	good	good	n/a	n/a
protein crystallization	poor	good	poor	moderate	n/a	n/a
bioculture	moderate	good	moderate	moderate	excellent, 3D	good, 3D
MARKET						
cost of production	high	medium	high	low	medium to high	low
reusability	yes	no	yes	yes	no	no
disposable device use	expensive	good	expensive	good	hard to store	good

a photosensitive glass can be considered as thermoset.

b most of the materials can be fabricated by laser ablation but compared with those obtained with lithographic or molding methods the ablated features usually have a rougher surface and are often misshaped.

c Excellent for Teflon.

d 1 Barrer = 10^−10^ [cm^3^ O_2_(STD)].cm.cm^−2^.s^−1^.cmHg^−1^.

e in the case of droplet microfluidics, biological or chemical reactions are confined to individual droplets, and the surface properties of the device material only affect the generation of the droplets.

Coatings and surfactants can further impact the surface characteristics and wettability of microfluidic systems. Modified natural and synthetic biosurfaces are used in diagnostics, biotherapeutics, tissue engineering, transplantation, immunology, oncology, drug targeting and release, biosensors, bioelectronics, and research and development. The biocompatibility of coatings depends on the supplier and application, as well as the substrate on which the coating is placed. Aspects of the manufacturing process, such as mold release and agents and cleaning compounds, also impact biocompatibility.

### 2.3 Testing at different stages of the product life cycle

#### 2.3.1 Early design and development testing

It is important to conduct leakage testing during the prototype and development stages of the device to understand the drawbacks of the design and make design iterations at an early stage. During the development phase, the aim of leak testing is mostly to identify the limits of mechanical strength, operating conditions and maximum allowable pressure for the device, either by using burst pressure or pressure decay methods (Bhagat et al., 2007; Eddings et al., 2008; Aran et al., 2010; Cortese et al., 2011; Casanova-Moreno et al., 2017; Borok et al., 2021); visual observation with dye or fluorescence and a pressure source (Chueh et al., 2007; Shiroma et al., 2016; Gonzalez-Gallardo et al., 2021) or gas sensor (Sparreboom et al., 2013); or quantification of the bond strength by tensile testing (Tang et al., 2006; Ouellet et al., 2010; Ren et al., 2015; Bakouche et al., 2020), blister, peeling, or razor blade tests. In establishing these tests, the dynamic conditions, as well as the location and sensitivity of the measurements, should be carefully considered. Leakage testing during the development phase helps to identify worst case operating scenarios of the system. To establish appropriate operating conditions, it may be beneficial to rapidly change flow rates during the testing to mimic a range of physiological environments. Real-time monitoring for leaks may also allow the developer to rapidly identify the source of the leak. During the early development stages, characterizing the extent of the leak is generally less critical than identifying whether a leak occurs. It is important to know the precise location of the leak, which can be elucidated using local tests with dyes or fluorospheres. By performing more rigorous, local testing during the early stages of development, the mechanisms behind the leaking, such as poor bonding or insufficient interconnections, can be determined and rectified.

#### 2.3.2 Final product testing

Final product testing is necessary to assess the performance and functionality of a device assembly in its final, pre-conditioned form. Issues occurring at the interfaces of components or due to manufacturing defects can be assessed by quality control testing. Burst pressure or visual leakage testing are destructive in nature, so they can be performed on a statistical sampling of units within a manufactured lot but not on every individual device. The sensitivity of final product testing is more critical than during the development phase and will be largely dependent on the detection methods used and their associated accuracies. Similarly, global tests are more applicable for final product testing compared to local tests. Global tests focus on identifying the presence of leak between the device and the outside environment (typically pressure decay test, bubble or sniffing tests) (Schroder, 2001). They can be conducted under static pressurized or dynamic flow conditions, and they should be conducted under real use operating conditions, considering the effect of parameters such as temperature and the chemical behavior of the real-life medium (e.g., blood). It is important to use statistical approaches to validate the number of test replicates to be performed to demonstrate safety with regards to final product leakage testing. Any novel leak test developed by a microfluidic device manufacturer can be appropriately validated by using different commercial devices and comparing the leakage test results to future transfer standards of similar devices to establish an acceptable level of safety and traceable performance.

#### 2.3.3 Product accessory testing

Oftentimes, manufacturers rely on third party accessories to support their devices throughout the product life cycle. This demand has created a market for third party manufacturers to specialize in fabricating specific components, such as micropumps, flow controllers, pressure sensors, and bubble traps. A survey among microfluidic device developers and manufacturers indicated that most companies either have no leakage specifications or unrealistic leakage specifications (e.g., zero tolerance) for their products (The Microfluidics Association, 2021). And the product labeling for these components rarely includes information on their leakage potential or maximum operating pressure. Nonetheless, leakage potential is dependent on how much the connectors are tightened, and the overall system performance is only as strong as the weakest component. When leakage is mentioned, more than often the details about the test protocol are not provided. For example, the labeling for a 4-way linear connector usually provides specifications about maximum pressure and temperature (Dolomite Microfluidics, 2022), while information about which interconnectors or tubing size to use in conjunction with their component is commonly lacking. The need for homogeneity in product specifications applies to valves as well. While microvalves are typically intended to be used for microfluidic applications with low flow rates (< 1 µl/min), operating specifications are sometimes provided in terms of the achievable burst pressure that does not cause breaks or cracks over a 1-min airtight test. Overall, the lack of details or referenced standard test protocols associated with microfluidic components make it difficult to validate claims regarding their safety and performance. In general, the device developer should select system components to use based on biocompatibility and mechanical integrity specifications, and then the final assembled device should be evaluated in its entirety for leakage since the individual components may perform differently depending on the operating conditions, the environment in which they are used, and how they are assembled into the final device.

### 2.4 Testing conditions

#### 2.4.1 Burst pressure testing

A burst test is a destructive method that is performed to determine the pressure at which a given component will “burst” or fail catastrophically. Burst pressure refers to the maximum internal pressure that a product can endure before it breaks. It is important to consider burst pressure when designing any sort of system, especially for products where failure is not allowable or in high pressure applications. Knowing the burst pressure allows manufacturers to create a sufficient safety factor to be applied in the product specifications. A burst test provides the margin between the maximum working pressure in the field and the pressure when the product completely fails. For safety reasons, burst pressure tests are either done with a liquid or gas in a containment chamber to fully contain the test apparatus for safety. For some biomedical applications such as for intravascular catheters, the safety factor is already specified in applicable standards or regulations (International Organization for Standardization, 2013).

To establish the burst pressure, the device is pressurized slowly, either continuously or stepwise, until the device fails, which is generally when a leak is detected. The burst pressure can be easily detected using real-time pressure monitoring or visual inspection. A practical way of doing this might be to increase the pressure by steps equivalent to 1/8 of the maximum pressure that the device is supposed to withstand. For instance, if the device is expected to operate up to a maximum pressure of 200 kPa, pressure would be increased using incremental steps of 25 kPa until failure. The duration at each pressure step depends on the specified minimal leak rate tolerated, the minimal detectable leak rate, and the intended duration of use of the device. Burst testing should be repeated with multiple samples, because oftentimes inconsistencies in the product will dictate this failure point.

#### 2.4.2 Testing at worst-case operating conditions

Testing at worst-case operating conditions is meant to provide some level of assurance that the device will function properly and as intended over its entire operating range, including at extreme conditions. For leakage testing, a 1.5 to 2 times pressure safety factor is typically applied during testing to demonstrate that the device will not fail even when subjected to pressures, temperatures, flow rates, and durations at or beyond their intended use. As for static pressure decay testing can be performed over the intended duration of use at 2/3 of the expected burst pressure, or at 1.5 to 2 times the operating pressure if the burst pressure is not known. This worst-case testing method is effective at identifying early “infant mortality” failures, which are unexpected and occur at the beginning of the product lifespan (Muhlbauer, 2004). These failures differ from other expected failures caused by, for instance, chemical or mechanical wear. Worst-case testing is normally conducted on a statistical sampling of the final products to confirm the structural stability of the devices after packaging, sterilization, simulated transportation, environmental exposure simulation, and accelerated or real-time storage.

#### 2.4.3 Vacuum-based testing

**FIGURE 3:**
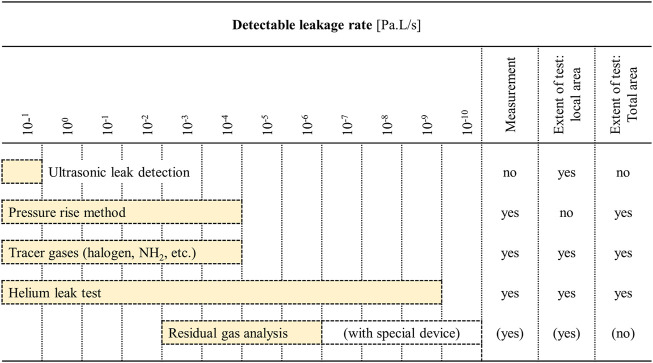
Applications and detection limits of various leak detection and leak tightness testing methods under vacuum. The detection limits specified here should only be regarded as reference values, since the true detection limits will depend on the conditions under with the test is performed. Adapted from Schroder (2001).

While positive pressure-based leak testing is perhaps most popular, vacuum can also be applied to the microfluidic system or its surroundings to determine the minimal pressure under which the device should operate. In the case of vacuum-based testing there are various approaches for leakage detection, such as ultrasonic leak detection, pressure rise method, and the use of different tracer gases. Figure 3 below shows the most popular vacuum-based approaches in increasing order of increasing test sensitivity (Schroder, 2001).

## 3 Knowledge gaps in leakage testing of microfluidic devices

Beyond the existing relevant standards identified in Tables 1, 4, there are at least three ISO technical committees with leakage testing action items: Technical Committee ISO/TC 135, *Non-destructive testing*, Technical Committee ISO/TC 138, *Plastic pipes, fittings, and valves for transport of fluids*, and Technical Committee ISO/TC 153, *Valves*. Some Subcommittees of ISO/TC 135, *Non-destructive testing* focus on leakage testing methods based on acoustic emissions to generate pressure differentials (International Organization for Standardization, 2016) and more generally on “leak testing” (Technical Committee ISO/TC 135, *Non-destructive testing*, Subcommittee SC 6, *Leak testing*). ISO/TC 135/SC 6 is dedicated to standardizing leakage testing in pressurized vessels and underground pipelines using radioactive tracer methods. Even though the application is far removed from the microfluidics domain, the ISO 20484:2017 standard, developed under the same Technical Committee ISO/TC 135, *Non-destructive testing*, includes pertinent vocabulary or nomenclature that could be universally used and adapted to describe leakage testing in microfluidic environments (International Organization for Standardization, 2017). It should be noted, however, that most of the definitions are related to gas-based detection methods.

**TABLE 4:** European Normative Standards related to non-destructive leak testing. The table is adapted from (Schroder, 2001).

Standard number	Title of the standard
EN 1330-8	Non-destructive testing - Terminology. Part 8: Terms used in leak tightness testing
EN 1518	Non-destructive testing - Leak Testing. Characterization of mass spectrometer leak detectors
EN 1779	Non-destructive testing - Leak Testing. Criteria for method and technique selection
EN 1593	Non-destructive testing - Leak Testing. Bubble emission techniques
EN 13184	Non-destructive testing - Leak Testing. Pressure change method
EN 13185	Non-destructive testing - Leak Testing. Tracer gas method
EN 13192	Non-destructive testing - Leak Testing. Calibration of gaseous reference leaks
EN 13625	Non-destructive testing - Leak Testing. Instructions for the selection of leak testing devices

The Technical Committee ISO/TC 138, *Plastic pipes, fittings, and valves for transport of fluids*, Subcommittee SC 5, *General properties of pipes, fittings and valves of plastic materials and their accessories -- Test methods and basic specifications*, is also developing test methods for leak tightness under negative pressure, internal pressure, or vacuum conditions. These protocols mostly use a vacuum or pressure gauge to identify leaks that are temperature dependent. The methods could be especially useful for testing interconnections because they require the presence of an elastomeric ring, which is commonly used to ensure leak tightness of microfluidic interconnections. Also, the Technical Committee ISO/TC 153, *Valves*, is dedicated to valves and has generated ISO 5208:2015 *Industrial valves*—*Pressure testing of metallic valves* (International Organization for Standardization, 2015). This standard combines aspects of two standards, EN12266-1 (European Standards, 2012) and API598 (American Petroleum Institute, 2009), and is a widely recognized reference in the fields of industrial valves and valves with soft seals.

Other standards organizations are involved in different types of leakage testing, some of which are shown in Table 4. In particular, the European standard DIN EN 1779 describes for the first time the criteria for methods and techniques selection for non-destructive leak testing (European Standards, 1999; Schroder, 2001), further assessed in other European Normative Standards. However, the general standards in Table 4, which were developed for non-destructive leak testing, have much larger leakage rates up to 12 orders of magnitude greater than leaks observed on the micro-scale. Thus, the small leakage rates and small sample volumes used in microfluidics are likely to require more sensitive measurement and detection technologies. To further emphasize this challenge, a methodology was only recently approved at the European Level to measure flow rates in microfluidic devices down to 0.0003 ml/h or 5 nL/min with 2.7% uncertainty (https://www.bipm.org). Not only is the leakage of small volumes very difficult to identify, but methods and techniques to reproducibly make these measurements are not yet available on the market.

Likewise, the Japanese Standards Association revised a standard for bubble leak testing (Japanese Standards Association, 2019), and ASTM and NASA have published standards for pressure decay leak tests (Dunlap et al., 2009; ASTM International, 2021a; ASTM International, 2021). Also, companies specializing in leak testing have proposed various leak testing evaluation systems and training modules (Hoffmann, 2012; Cincinnati Test Systems, 2014; Stering et al., 2014; ATEQ, 2020; Dewailly, 2020; HeMaTech, 2021; Blue Sky Biomedical, 2022; TQC Ltd, 2022). Despite some progress made in these general activities and in other more established fields, there are currently no dedicated leakage testing and detection standards for microfluidic systems or evidence that existing standards for other applications can be used for microfluidic devices, given the considerable difference in measurement ranges and total volumes.

## 4 Classification of leakage testing based on application

**FIGURE 4:**
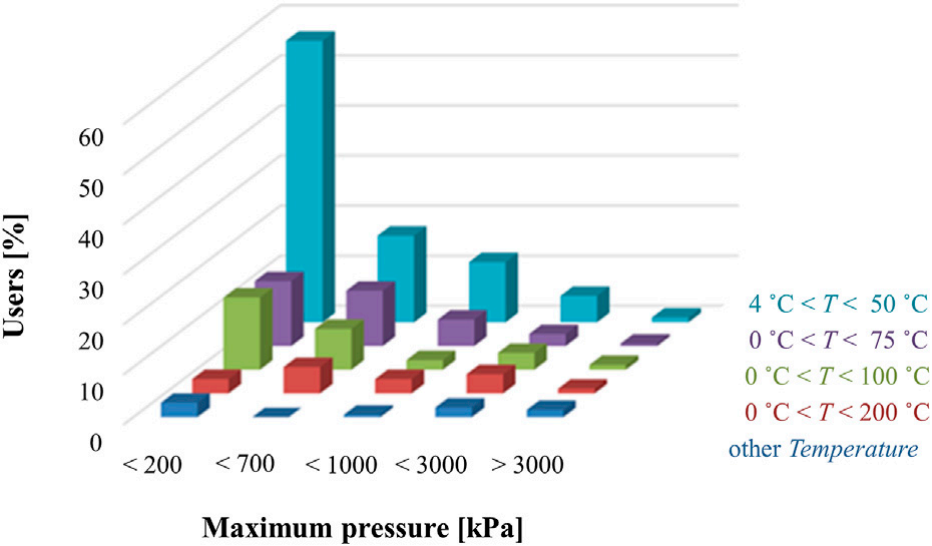
Distribution of microfluidic applications based on the operating temperature and pressure classes (The Microfluidics Association, 2016).

**FIGURE 5:**
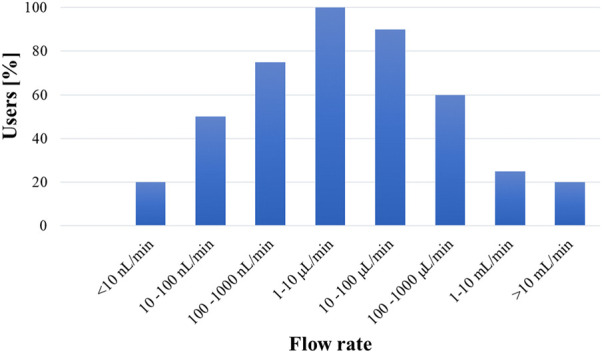
Typical flow rates used in microfluidic devices based on industry feedback (The Microfluidics Association, 2016).

One of the biggest challenges in the definition of guidelines, protocols and standards for the development and use of microfluidic products is the wide range of applications, technologies, media used, and operating conditions. The type of application dictates parameters such as the flow rate, pressure, and temperature of the microfluidic system, making it difficult to define generic test protocols to assist in the development and test of more reliable microfluidic devices. Based on feedback from key stakeholders through an industry-wide survey that includes developers, users, and suppliers (The Microfluidics Association, 2016) a preliminary attempt at classifying microfluidic products in terms of operating conditions is collected in Table 5. Figures 4, 5 additionally summarize key physical characteristics and physical limitations of currently used devices (The Microfluidics Association, 2015). The classifications are made based on pressure, temperature and flow rate and are proposed to provide manufacturers and consumers with tangible expectations of device capabilities and achievable operating ranges.

**TABLE 5:** These proposed application classes based on operating conditions have been adapted from ISO 22916:2022 (International Organization for Standardization, 2022).

Class	Maximum pressure [kPa]	Maximum temperature [°C]	Minimum temperature [°C]
Capillary devices	---	50	4
PT 200/50	200	50	4
PT 200/75	200	75	4
PT 200/100	200	100	4
PT 700/50	700	50	4
PT 700/100	700	100	4
PT 3000/50	3,000	50	4

The proposed temperature and pressure ranges in Table 5 were chosen based on the following considerations: (i) the temperature range of 4°C to 50°C is used for off-the-shelf pumps; (ii) the temperature range from 4°C to 100°C encompasses applications such as polymerase chain reaction (PCR); (iii) as some suppliers were concerned that the difference between 50 and 100°C was too large to accurately represent a variety of device applications, 75°C was chosen as an intermediate upper limit; (iv) the maximum pressure values of 200 kPa and 700 kPa are commonly generated by the flow regimes of typical microfluidic products. Retrospectively, an additional ‘capillary devices’ category was added to better capture this underrepresented microfluidic device type.

While most microfluidic products operate within application class PT 200 kPa/50°C (Figure 4), some applications run at higher pressures and temperatures than those listed in Table 5. For example, the current classification system should be extended to high-pressure microfluidic applications, such as microfluidic based chromatographers (Yin and Killeen, 2007) or microreactors (Tiggelaar et al., 2007; Marre et al., 2010), which function in the megapascal range. Additionally, to better understand the flow conditions under which most microfluidic devices operate, Figure 5 shows what the community experts view as typical flow rates used in microfluidics technologies (The Microfluidics Association, 2016). Overall, the consensus among the community is that most microfluidic devices use flow rates ranging from 10 nl/min up to 1 ml/min.

**FIGURE 6:**
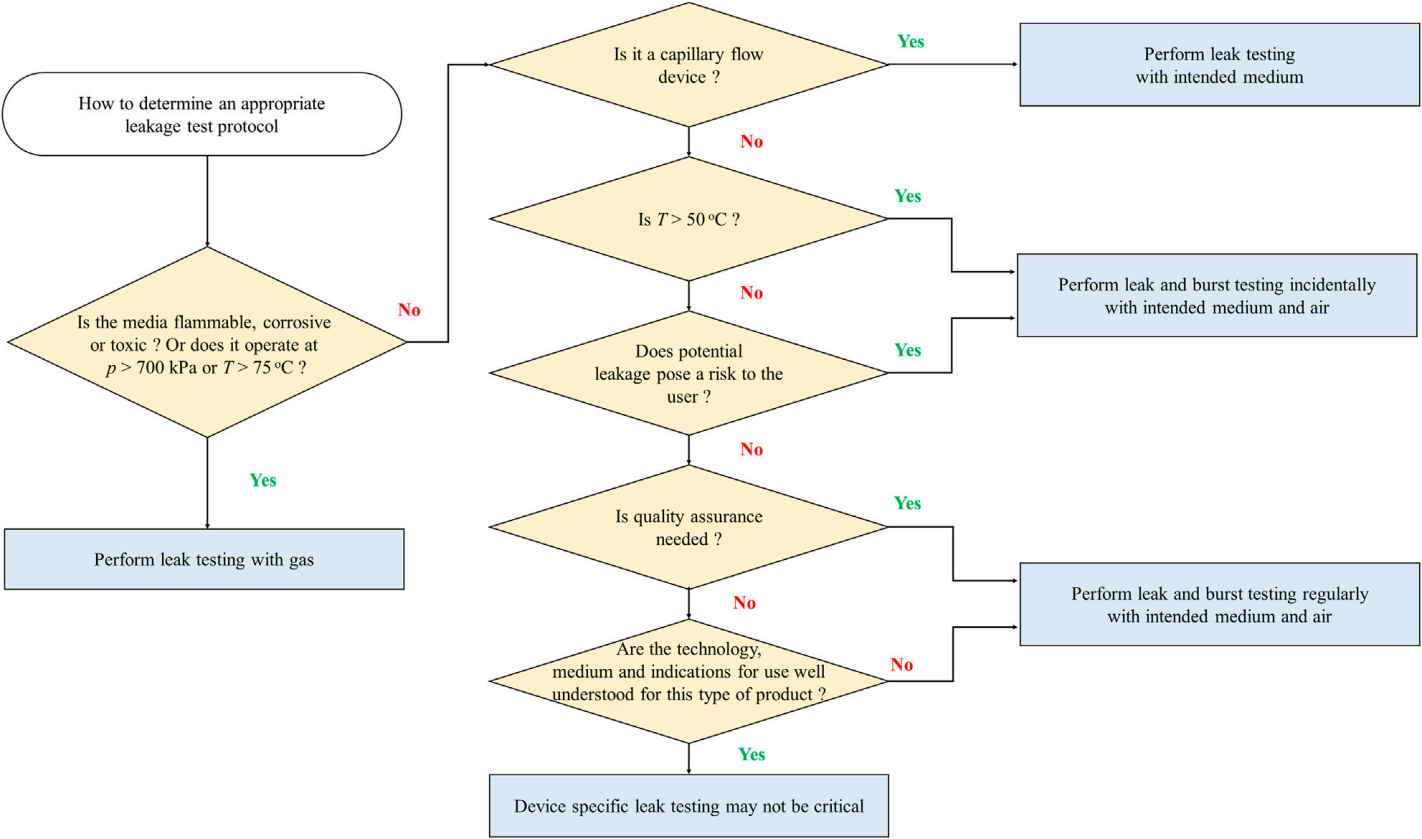
Use of classification criteria to select an appropriate leakage test protocol. Adapted from van Heeren et al. (2022).

The proposed classifications are an important first step towards developing industrywide working classes to define microfluidics testing strategies, methods and reliability models standard test protocols. Understanding these key limitations also helps the community to identify practical, device-specific approaches for determining the flow conditions under which leaking may occur. Nonetheless, given the diversity of microfluidic products, it is challenging and not necessarily the best approach to develop a single leakage test that fits all cross-cutting applications. Test protocols should be flexible to account for criteria such as the type of media used, flow conditions, complexity of the device, and the risk to the user. To help in the selection of the most appropriate procedure, the microfluidics community has begun to develop a flowchart (Figure 6) (van Heeren et al., 2022) to help users select an appropriate leakage test protocol based on the following considerations: pressure, temperature, risk to the operator or the environment, and the need for quality assurance control. In general, it would be most beneficial to the community to first develop tests for the most common device types that operate with water-based media at temperatures between 4°C and 50°C, pressures below 200 kPa, and flow rates between 1 μ L/min and 1 ml/min. Specific test protocols described by van Heeren et al. (2022) can then be used in various sub-ranges of these systems. Once test protocols are established for the most common microfluidic device classes, additional procedures can then be adapted or extrapolated to assess other application classes. Note that the protocols referenced in Figure 6 are still under development to support common microfluidic applications.

## 5 Discussion

Industry experts representing a range of microfluidic applications have indicated testing challenges the community encounters when fabricating microfluidic-based devices (The Microfluidics Association, 2015). One of the most important issues is the lack of harmonization in vocabulary needed to understand and communicate common microfluidic terms (Reyes et al., 2020; van Heeren et al., 2022a). Another challenge is that there are no standard tests used to evaluate microfluidic devices. It is difficult to quickly and accurately measure parameters such as flow, length, and volume on the microscale, especially as the microfluidics field tries to bridge the gap between laboratory-based prototypes and high-volume manufactured end products. Leaks are one of the most common failure modes in microfluidic-based devices. While there are ways to assess leakage potential on the bench prior to commercial use of the microfluidic system, their suitability, sensitivity and accuracy are not well understood.

Regardless of the device application, it is important to understand if and how much a device leaks by qualitatively or quantitatively determining leakage rates. These means of characterization can be used to demonstrate safety and performance of microfluidic systems. The leakage detection limits will vary depending on the sensitivity of the detection instrumentation and the physical conditions under which the test is performed. Leakage measurements made using a well-controlled benchtop setup are likely to be more accurate and reliable than those made in a dynamic environment in the field. The duration of the leakage test can directly impact the test sensitivity. For substantial leaks, the leakage test duration may be short if the pressure decay is significant, whereas testing may be needed over the intended duration of use of the end-product if leaking is more difficult to detect.

Leaks are impacted by the microchannel design, the properties of the media flowing through the device, and surface interactions. One particular challenge with microfluidics is that the feature sizes can range from nanometers to hundreds of microns. Manufacturing issues and deviations from the intended channel dimensions may impact the flow resistivity, thereby affecting the performance of the device. Since surface tension is inversely proportional to channel length, surface tension effects are exacerbated in microfluidic channels. Different materials at the interconnections of these devices add complexity to the system (van Heeren et al., 2022). Furthermore, wettability of the media-contacting interface can be impacted by the topography of the surface as well as the chemical nature of the fluid used. Elastomeric substrates such as PDMS can induce air bubbles within the channels, which may hinder device performance and complicate leakage testing. When considering the media to use for leakage testing, the benefits of non-destructive testing along with increased sensitivity with gases need to be compared against the benefits of using application-specific liquids and the additional safety precautions required for handling gases. Another variable to consider for non-destructive testing is the pressure to which the system is subjected. Testing a device at a higher pressure can overstress the system, which may accelerate mechanical integrity failures of the device and exacerbate leaking.

Microfluidic devices are unique in that they often rely on hybridization strategies during fabrication to assemble different parts into the end-product (Silverio and Cardoso, 2021). The ability to form bonded interfaces as well as the overall component reliability depends on material properties of the microfluidic components, such as stress, toughness, elasticity, ductility, or plasticity. The bonding must be strong enough to withstand the expected pressure and should remain leakage-free throughout the duration of use. If the bonding or interface strength is much lower than the structural strength of the material, leakage will ultimately occur, even at moderate pressures (Eddings et al., 2008; Cortese et al., 2011). Variations in temperature, which cause contractions and expansions of materials, can result in alterations of the physical dimensions of bonded parts or can alter the leak path dimensions. This becomes more relevant if the device is exposed to thermal cycles, as is the case of microfluidic chips being used for PCR (polymerase chain reaction) testing, microfluidic heat exchangers or microfluidic reactors for chemistry. The quality of the polymer used, glass transition temperature, melting temperature, molecular weight and distribution can all impact the strength of the bond. The bond strength can be measured by adhering the test material to an interface and then by applying force, but it is a destructive and time-consuming process. An alternative is to simply pressurize the device with air and check for leakage. Contaminants and voids can then be identified at the bonding sites. Given the importance visualization plays in leak detection, it is also useful to have bonds and device components that are translucent and smooth to enable optical access to the bond sites for inspection.

The duration of a leakage test should be chosen based on the intended duration of device use of the final device for its specific application. Leaks in fast diagnostic tests do not need to be evaluated for as long a duration as those microfluidic systems used for longer, continuous processing applications. It may be possible to reduce the testing time by performing the test under worst-case conditions such as higher pressures or temperatures. Manufacturers should determine what constitutes the worst-case performance or design parameters for the device and then perform leakage testing at those conditions. Prolonged testing periods should also include pressurization or depressurization or deliberate fluctuations in pressure if the device is expected to encounter those scenarios during real-life use to understand how the summation of these factors influences the leakage rate. Test temperature can also impact the duration of testing, bulk flow rate through the channel, leakage rate, and fluid properties such as surface tension. During leakage testing, the temperature should be carefully controlled. Typically, the leakage test temperature should be the same as the temperature at which the commercial product operates.

Leakage testing prior to commercial use should be performed on the final, preconditioned product, taking into consideration the effects of storage conditions, sterilization, transport, environmental exposure, accelerated aging or real time aging on the mechanical integrity of the device. While reusable devices are outside the scope of this review, leak testing is also beneficial for reusable systems to demonstrate that they will not leak when subjected to multiple uses and reprocessing. Lastly, the number of replicate samples depends on the frequency of device failures, the complexity of the device, the types of failures, and the consequences of failure for the user. Depending on the type of device, and the risk to the user from failure, a high confidence level requiring large sample sizes may be required.

## 6 Conclusions and outlook

There is a need for simple, standardized test methods in microfluidics. Recent activities to develop leakage test methods, such as the collaboration of the Microfluidics Association (MFA) with MFMET (Establishing metrology standards in microfluidic devices, mfmet.eu)-a project financed by the European Metrology Program for Innovation and Research (EMPIR) to define metrology challenges for microfluidics, are a step in the right direction towards improving the safety and performance of microfluidic devices. Through this project an action plan was formulated for industry, and the following questions should be considered when developing appropriate device-specific leakage testing:

1) What is the minimum amount of liquid that can be detected by visual inspection? Can this detection limit be improved by using markers or tracers?
2) What is the minimum amount of gas that can be detected during gas-based leakage testing? Can this detection limit be improved by using different measurement tools?
3) Can the gas leakage simulate or correlate to liquid leakage of the same system? What is the accuracy of gas-vs. liquid-based measurements of pressure loss across the system?
4) What is the accuracy of flow rate measurements at the inlet and outlet of the system?
5) What are the failure modes and locations of leakage testing?
6) Is it possible to modify test parameters to accelerate the detection of leaks during testing?
7) How do temperature and pressure impact leakage test results?

The community must work together towards development of consensus standards with the objective of improving device safety and reliability (Reyes et al., 2020). This paper focuses on a critical area of need for protocol development in leakage testing. While such efforts are currently underway, much progress is still needed to promote wider adoptability of simple leakage testing protocols capable of achieving predetermined specifications. It was shown that microfluidic devices typically operate within the broad flow rate range of 10 nl/min to 1 ml/min (Figure 5), with most applications in the narrower 1 μ L/min to 100 μ L/min flow rate range. Therefore, leakage testing methods for microfluidics-based products should encompass this range. Some experts in the community are reluctant to adapt standard liquid-based tests because of the low sensitivity. While gas-based tests are more sensitive and non-destructive, most microfluidic applications use liquids, so both types of tests have utility in validating microfluidic-based devices. In any case, these methods are not validated for such low flows and no validated standard methods in this flow regime are currently available. Metrology labs can measure these low flows, but the gap between their capabilities and practical methods suitable for industry-wide use remains wide. A way to bridge this gap is to develop leakage test models, conduct interlaboratory studies to validate and calibrate leakage test methods, and establish consensus based standard microfluidic leakage tests. The design of transfer standards should consider the wide varieties of applications seen in microfluidics and reflect the interest of multiple stakeholders. Given the consistent growth of microfluidics-based industries and the time needed to develop international consensus standards, we hope that the community can come together to actively pursue a common goal.

Given the diversity of the microfluidics fields and the disparity in sensitivity across the wide range of applications, it may be beneficial to classify microfluidic systems based on application type and specifications to perform appropriate leakage testing. The classification schemes discussed in this review article are a starting point for having a stratified approach towards standardized leakage test development for microfluidics. Further classification can be made based on flow rate, media used (chemicals or biologicals), flammability, corrosiveness, and toxicity of media. Complex surface effects including surface tension particularly for liquids, multiphase flow behavior, non-Newtonian flow behavior may also be considered. Academia, industry, and regulators should continue to collaboratively work in these areas to address and unmet need and develop standard test protocols that will account for the many factors affecting leakage in microfluidic systems.

## Data Availability

The original contributions presented in the study are included in the article; further inquiries can be directed to the corresponding author.

## References

[B1] Adegboye M. A. Wai-Keung F. Karnik A. (2019). Recent advances in pipeline monitoring and oil leakage detection technologies: Principles and approaches. Sensors 19 (11), 2548. 10.3390/s19112548 PMC6603558 31167413

[B2] American Petroleum Institute (2009). API 598:2009. Valve inspection and testing. Available at: https://www.api.org:443/(Accessed January 23, 2022).

[B3] Amesz J. (1966). Conversion of leak flow-rates for various fluids and different pressure conditions. EUR 2982. Eur. At. Energy Community 20, 33455.

[B4] Antelius M. Fischer A. C. Niklaus F. Stemme G. Roxhed N. (2011). “Hermetic integration of liquids in MEMS by room temperature, high-speed plugging of liquid-filled cavities at wafer level,” in Proceedings of the 2011 IEEE 24th International Conference on Micro Electro Mechanical Systems, 23-27 January 2011 (Cancun, Mexico, 356–359. 10.1109/MEMSYS.2011.5734435

[B5] Araci I. E. Su B. Quake S. R. Mandel Y. (2014). An implantable microfluidic device for self-monitoring of intraocular pressure. Nat. Med. 20 (9), 1074–1078. 10.1038/nm.3621 25150497

[B6] Aran K. Sasso L. A. Kamdar N. Zahn J. D. (2010). Irreversible, direct bonding of nanoporous polymer membranes to PDMS or glass microdevices. Lab. Chip 10 (5), 548. 10.1039/b924816a 20162227 PMC4538600

[B7] ASTM International (2021). Astm E2930-13(2021) - standard practice for pressure decay leak test method. Available at: https://www.astm.org/e2930-13r21.html.

[B8] ASTM International (2021a). Astm F2095-07(2021) - standard test methods for pressure decay leak test for flexible packages with and without restraining plates. Available at: https://www.astm.org/f2095-07r21.html.

[B9] ATEQ (2020). Air leak testing methods - air decay, differential, mass flow. Available at: https://www.ateq-nl.com/wp-content/uploads/2021/04/ATEQ-drukval-verschilfdruk-of-massflow.pdf (Accessed January 23, 2022).

[B10] Bakouche M. T. Ganesan S. Guérin D. Hourlier D. Bouazaoui M. Vilcot J. P. (2020). Leak-free integrated microfluidic channel fabrication for surface plasmon resonance applications. J. Micromech. Microeng. 30 (12), 125003. 10.1088/1361-6439/abb991

[B11] Batista E. Furtado A. Ferreira M. C. Godinho I. Álvares M. Afonso J. (2021). Uncertainty calculations in optical methods used for micro flow measurement. Meas. Sensors 18, 100155. 10.1016/j.measen.2021.100155

[B12] Bhagat A. A. Jothimuthu P. Pais A. Papautsky I. (2007). Re-usable quick-release interconnect for characterization of microfluidic systems. J. Micromech. Microeng. 17 (1), 42–49. 10.1088/0960-1317/17/1/006

[B13] Blue Sky Biomedical (2022). How does the leak test work? Available at: https://blueskybiomedical.com/how-does-the-leak-test-work/(Accessed January 23, 2022).

[B14] Borók A. Laboda K. Bonyár A. (2021). PDMS bonding technologies for microfluidic applications: A review. Biosensors 11 (8), 292. 10.3390/bios11080292 34436094 PMC8394141

[B15] Casanova-Moreno J. To J. Yang C. W. T. Turner R. F. B. Bizzotto D. Cheung K. C. (2017). Fabricating devices with improved adhesion between PDMS and gold-patterned glass. Sensors Actuators B Chem. 246, 904–909. 10.1016/j.snb.2017.02.109

[B16] Chueh B.-h. Huh D. Kyrtsos C. R. Houssin T. Futai N. Takayama S. (2007). Leakage-free bonding of porous membranes into layered microfluidic array systems. Anal. Chem. 79 (9), 3504–3508. 10.1021/ac062118p 17388566 PMC2517097

[B17] Cincinnati Test Systems (2014). How to establish an acceptable leak rate. Application bulletin: #120. Available at: https://www.cincinnati-test.com/images/AB120%20Setting%20Acceptable%20Test%20Criteria.pdf (Accessed January 23, 2022).

[B18] Cincinnati Test Systems (2020). How to leak and blockage test microfluidic chips or cassettes. Application note ▪ medical. Available at: https://www.cincinnati-test.com/documents/PDFs/Leak-blockage-test-microfluidic-chips.pdf (Accessed February 09, 2022).

[B19] Cortese B. Mowlem M. C. Morgan H. (2011). Characterisation of an irreversible bonding process for COC–COC and COC–PDMS–COC sandwich structures and application to microvalves. Sensors Actuators B Chem. 160 (1), 1473–1480. 10.1016/j.snb.2011.07.040

[B20] Dewailly A.-M. (2020). Setting an air leak testing - quality control specification. Leak Testing Academy. ATEQ. Available at: https://atequsa.com/wp-content/uploads/2020/09/setting-testing-specs.pdf (Accessed January 23, 2022).

[B21] Dolomite Microfluidics (2022). Linear connector. (no date). Available at: https://www.dolomite-microfluidics.com/product/linear-connector (Accessed January 23, 2022).

[B22] Dunlap P. H. Daniels C. C. Wasowski J. L. Garafolo N. G. Penney N. Steinetz B. M. (2009). “Pressure decay testing methodology for quantifying leak rates of full-scale docking system seals. Technical memorandum,” in Propulsion Conference & Exhibit, Denver, Colorado, USA, August 2–5, 2009. Available at: https://ntrs.nasa.gov/api/citations/20100019164/downloads/20100019164.pdf. 10.2514/6.2009-5319

[B23] Eddings M. A. Johnson M. A. Gale B. K. (2008). Determining the optimal PDMS–PDMS bonding technique for microfluidic devices. J. Micromech. Microeng. 18 (6), 067001. 10.1088/0960-1317/18/6/067001

[B24] European Standards (2012). DIN EN 12266-1 Industrial valves - testing of metallic valves - Part 1: Pressure tests, test procedures and acceptance criteria - mandatory requirements. Available at: https://www.en-standard.eu/din-en-12266-1-industrial-valves-testing-of-metallic-valves-part-1-pressure-tests-test-procedures-and-acceptance-criteria-mandatory-requirements/(Accessed January 23, 2022).

[B25] European Standards (1999). DIN EN 1779 Non-destructive testing - leak testing - criteria for method and technique selection. Available at: https://www.en-standard.eu/din-en-1779-non-destructive-testing-leak-testing-criteria-for-method-and-technique-selection/.

[B26] German Institute for Standardization (1999). DIN EN 1779:1999. Non-destructive testing - leak testing - criteria for method and technique selection. Available at: https://www.din.de/en/getting-involved/standards-committees/nmp/publications/wdc-beuth:din21:19709150 (Accessed May 17, 2022).

[B27] Gonzalez-Gallardo C. L. Díaz A. D. Casanova-Moreno J. R. (2021). Improving plasma bonding of PDMS to gold-patterned glass for electrochemical microfluidic applications. Microfluid. Nanofluidics 25, 20. 10.1007/s10404-021-02420-3

[B28] Health Executive Safety (2013). Control of substances hazardous to health. ISBN 9780717665822. 6th ed. New York, USA: Crown. Available at: https://www.hse.gov.uk/pubns/books/l5.htm.

[B29] HeMaTech(2021). Leak testing. Available at: https://www.hematech.de/en/leak-testing.html (Accessed January 23, 2022).

[B30] Hoffmann J. (2012). Leak testing 101 - Part 1. Skokie: InterTech Development Company. Available at: https://intertechdevelopment.com/2012/leak-testing-101-part-1 (Accessed January 23, 2022).

[B31] Hu K. Yu F. Ho T.-Y. Chakrabarty K. (2014). Testing of flow-based microfluidic biochips: Fault modeling, test generation, and experimental demonstration. IEEE Trans. Comput. -Aided. Des. Integr. Circuits Syst. 33 (10), 1463–1475. 10.1109/TCAD.2014.2336215

[B32] International Organization for Standardization (2013). ISO 10555-1:2013+A1:2017 Intravascular catheters. Sterile and single-use catheters – Part 1: General requirements. Available at: https://www.iso.org/standard/54884.html.

[B33] International Organization for Standardization (2016). ISO 18081:2016 Non-destructive testing — acoustic emission testing (AT) — leak detection by means of acoustic emission. Available at: https://www.iso.org/standard/61326.html.

[B34] International Organization for Standardization (2017). ISO 20484:2017 non-destructive testing — leak testing — vocabulary. Available at: https://www.iso.org/standard/68188.html.

[B35] International Organization for Standardization (2022). ISO 22916:2022 Microfluidic devices — interoperability requirements for dimensions, connections and initial device classification. Available at: https://www.iso.org/standard/74157.html.

[B36] International Organization for Standardization (2015). ISO 5208:2015 Industrial valves — pressure testing of metallic valves. Available at: https://www.iso.org/standard/65111.html.

[B37] Japanese Standards Association (2019). Jis Z 2329:2019 - non-destructive testing - methods for bubble leak testing. Available at: https://webdesk.jsa.or.jp/books/W11M0090/index/?bunsyo_id=JIS+Z+2329%3A2019 (Accessed May 24, 2022).

[B38] Kauffman P. Fu E. Lutz B. Yager P. (2010). Visualization and measurement of flow in two-dimensional paper networks. Lab. Chip 10, 2614–2617. 10.1039/C004766J 20676410 PMC4892119

[B40] Limitless Shielding (2020). Gas permeable PDMS membranes. Available at: https://limitless-shielding.com/gas-permeable-pdms-membranes/?gclid=EAIaIQobChMIgvCL9OGl-QIVAJBoCR3wtAwMEAMYASAAEgK3IPD_BwE (Accessed July 29, 2022).

[B41] Liu X. Li S. (2014). An electromagnetic microvalve for pneumatic control of microfluidic systems. SLAS Technol. 19 (5), 444–453. 10.1177/2211068214531760 24742860

[B43] Marre S. Adamo A. Basak S. Aymonier C. Jensen K. F. (2010). Design and packaging of microreactors for high pressure and high temperature applications. Ind. Eng. Chem. Res. 49 (22), 11310–11320. 10.1021/ie101346u

[B44] Muhlbauer W. K. (2004). in Risk: Theory and application. Pipeline risk management manual. Editor Muhlbauer W. K. (Houston, Texas: Gulf Professional Publishing), 1-19. ScienceDirect. 10.1016/B978-075067579-6/50004-2

[B45] Mukhopadhyay R. (2007). When PDMS isn’t the best. Anal. Chem. 79 (9), 3248–3253. 10.1021/ac071903e 17523228

[B47] Ogheard F. Cassette P. Boudaoud A. W. (2020). Development of an optical measurement method for “sampled” micro-volumes and nano-flow rates. Flow Meas. Instrum. 73, 101746. 10.1016/j.flowmeasinst.2020.101746

[B48] Oosterbroek R. E. Hermes D. C. Kakuta M. Benito-Lopez F. Gardeniers J. G. E. Verboom W. (2005). Fabrication and mechanical testing of glass chips for high-pressure synthetic or analytical chemistry. Microsyst. Technol. 12 (5), 450–454. 10.1007/s00542-005-0043-5

[B49] Ouellet E. Yang C. W. T. Lin T. Yang L. L. Lagally E. T. (2010). Novel carboxyl-amine bonding methods for poly (dimethylsiloxane)-based devices. Langmuir 26 (14), 11609–11614. 10.1021/la1012582 20575547

[B50] Real Zero (2009). Guide to good leak testing. Refrigerant emissions and leakage ZERO project. Available at: https://www.epa.gov/sites/default/files/documents/RealZeroGuidetoGoodLeakTesting.pdf (Accessed February 09, 2022).

[B51] Ren K. Zhou J. Wu H. (2013). Materials for microfluidic chip fabrication. Acc. Chem. Res. 46 (11), 2396–2406. 10.1021/ar300314s 24245999

[B52] Ren Y. Huang S. H. Mosser S. Heuschkel M. O. Bertsch A. Fraering P. C. (2015). A simple and reliable PDMS and SU-8 irreversible bonding method and its application on a microfluidic-MEA device for neuroscience research. Micromachines 6 (12), 1923–1934. 10.3390/mi6121465

[B53] Reyes D. R. van Heeren H. Guha S. Herbertson L. Tzannis A. P. Ducrée J. (2020). Accelerating innovation and commercialization through standardization of microfluidic-based medical devices. Lab. Chip 21, 9–21. 10.1039/D0LC00963F 33289737

[B54] Sagi H. (2001). Advanced leak test methods. Available at: https://www.assemblymag.com/articles/83578-advanced-leak-test-methods (Accessed February 09, 2022).

[B55] Salvo P. Hernandez-Silveira M. (2016). Wireless medical systems and algorithms: Design and applications. New York. USA: CRC Press. Taylor and Francis Group, LLC. ISBN-13: 978-1-4987-0078-8 (e-book).

[B56] Schroder G. (2001). New European standard for the selection of a suitable method for leak detection and leak tightness testing European standard EN 1779, 1999. Available at: https://core.ac.uk/download/pdf/157744013.pdf .

[B57] Sen A. K. Darabi J. Knapp D. R. (2009). Design, fabrication and test of a microfluidic nebulizer chip for desorption electrospray ionization mass spectrometry. Sensors Actuators B Chem. 137 (2), 789–796. 10.1016/j.snb.2009.02.002 PMC2682712 20161284

[B58] Shen F. Ai M. Ma J. Li Z. Xue S. (2020). An easy method for pressure measurement in microchannels using trapped air compression in a one-end-sealed capillary. Micromachines 11, 914. 10.3390/mi11100914 PMC7650790 33008031

[B59] Shiroma L. S. Piazzetta M. H. O. Duarte-Junior G. F. Coltro W. K. T. Carrilho E. Gobbi A. L. (2016). Self-regenerating and hybrid irreversible/reversible PDMS microfluidic devices. Sci. Rep. 6 (1), 26032–26112. 10.1038/srep26032 27181918 PMC4867595

[B60] Silverio V. Cardoso S. (2021). “Lab-on-a-chip: Systems integration at the microscale,” in Drug delivery devices and therapeutic systems. Editor Chappel E. (Amsterdam, Netherlands: Elsevier Science). 10.1016/B978-0-12-819838-4.00020-1

[B61] Sparreboom W. van de Geest J. Katerberg M. Postma F. Haneveld J. Groenesteijn J. (2013). Compact mass flow meter based on a micro coriolis flow sensor. Micromachines 4, 22–33. 10.3390/mi4010022

[B62] Stering M. Dahlberg M. Adams T. de Wilde D. Fenge C. BioProcess International (2014). Pressure decay method for postinstallation single-use bioreactor bag testing. Available at: https://bioprocessintl.com/upstream-processing/upstream-single-use-technologies/pressure-decay-method-postinstallation-single-use-bioreactor-bag-testing/(Accessed January 23, 2022).

[B63] Tang K. C. Liao E. Ong W. L. Wong J. D. S. Agarwal A. Nagarajan R. (2006). Evaluation of bonding between oxygen plasma treated polydimethyl siloxane and passivated silicon. J. Phys. Conf. Ser. 34 (1), 155–161. 10.1088/1742-6596/34/1/026

[B64] The Microfluidics Association (2021). Results from a leakage survey. Retrieved from. Available at: https://microfluidics-association.org.

[B65] The Microfluidics Association (2016). Survey on Microfluidic test guidelines. Retrieved from. Available at: https://microfluidics-association.org.

[B66] The Microfluidics Association (2015). Survey on reliability of microfluidics-based devices and components. Retrieved from. Available at: https://microfluidics-association.org.

[B67] Tiggelaar R. M. Benito-López F. Hermes D. C. Rathgen H. Egberink R. J. M. Mugele F. G. (2007). Fabrication, mechanical testing and application of high-pressure glass microreactor chips. Chem. Eng. J. 131 (1–3), 163–170. 10.1016/j.cej.2006.12.036

[B68] TQC Ltd (2022). Leak testing. (no date). Available at: https://www.tqc.co.uk/our-services/leak-testing/(Accessed January 23, 2022).

[B69] Trietsch S. J. Naumovska E. Kurek D. Setyawati M. C. Vormann M. K. Wilschut K. J. (2017). Membrane-free culture and real-time barrier integrity assessment of perfused intestinal epithelium tubes. Nat. Commun. 8 (1), 262. 10.1038/s41467-017-00259-3 28811479 PMC5557798

[B70] Vacuum Pfeiffer (2018). Micro-flow leak testing. Available at: https://www.pfeiffer-vacuum.com/filepool/file/literature/micro-flow-leak-testing-atc-pl0017pen.pdf?referer=2250 (Accessed February 09, 2022).

[B71] van Heeren H. Davies M. Keiser A. Lagrauw R. Reyes D. R. Silverio V. (2022). Protocols for leakage testing [White paper]. Retrieved from. Available at: https://microfluidics-association.org. 10.5281/zenodo.6602161

[B72] van Heeren H. Silverio V. Pecnik C. Batista E. (2022a). Metrology challenges for microfluidics. Commercial micro manufacturing magazine. Available at: http://www.cmmmagazine.com/cmm-articles/metrology-challenges-for-microfluidics (Accessed May 09, 2022).

[B73] Vowell S. (2009). Microfluidics: The effects of surface tension. Available at: http://citeseerx.ist.psu.edu/viewdoc/download?doi=10.1.1.509.7902&rep=rep1&type=pdf (Accessed February 09, 2022).

[B74] Vtech (2006). Leak detection theory and practice - comparison among leak testing techniques. Available at: https://www.vtechonline.com/products/leak-testing (Accessed February 09, 2022).

[B75] Wang J. Zheng M. Wang W. Li Z. (2010). Optimal protocol for molding PDMS with a PDMS master – chips and Tips. Available at: https://blogs.rsc.org/chipsandtips/2010/07/06/optimal-protocol-for-moulding-pdms-with-a-pdms-master/?doing_wp_cron=1653772062.1807069778442382812500.

[B76] Xu B.-Y. Yan X.-N. Xu J.-J. Chen H.-Y. (2012). One step high quality poly (dimethylsiloxane)-hydrocarbon plastics bonding. Biomicrofluidics 6 (1), 16507. 10.1063/1.3694251 22685512 PMC3370403

[B77] Yang L.-J. Kao A-F. (2010). “Gas permeation in PDMS monitored by on-site pressure sensors,” in IEEE 5th International Conference on Nano/Micro Engineered and Molecular Systems, Xiamen, China, 20-23 January 2010. 10.1109/nems.2010.5592233

[B78] Yin H. Killeen K. (2007). The fundamental aspects and applications of Agilent HPLC-Chip. J. Sep. Sci. 30, 1427–1434. 10.1002/jssc.200600454 17623422

[B79] Yin Z. Zou H. (2018). A fast and simple bonding method for low cost microfluidic chip fabrication. J. Electr. Eng. 69 (1), 72–78. 10.1515/jee-2018-0010

[B80] Zaman D. Tiwari M. K. Gupta A. K. Sen D. (2019). A review of leakage detection strategies for pressurised pipeline in steady-state. Eng. Fail. Anal. 109, 104264. 10.1016/j.engfailanal.2019.104264

[B81] Zhang J. Han P. Twomey M. (2019). Overview of pipeline leak detection technologies. Available at: https://asgmt.com/wp-content/uploads/Papers/Overview%20of%20Pipeline%20Leak%20Detection%20Technologies.pdf (Accessed February 09, 2022).

